# Prototype foamy virus downregulates RelB expression to facilitate viral replication

**DOI:** 10.1002/2211-5463.12968

**Published:** 2020-09-15

**Authors:** Junshi Zhang, Chenchen Wang, Xiaopeng Tuo, Keli Chai, Yali Xu, Wentao Qiao, Juan Tan

**Affiliations:** ^1^ Key Laboratory of Molecular Microbiology and Technology Ministry of Education College of Life Sciences Nankai University Tianjin China

**Keywords:** IP, LTR, prototype foamy virus, RelB, transcription

## Abstract

Foamy viruses (FVs) are classified in the subfamily *Spumaretrovirinae* and bridge the gap between *Orthoretrovirinae* and *Hepadnaviridae*. FVs have strong cytopathic effects against cells cultured *in vitro*. However, they establish lifelong latent infections without evident pathology in the host. The roles of cellular factors in FV replication are poorly understood. To better understand this area, we determined the transcriptomes of HT1080 cells infected with prototype foamy virus (PFV) to measure the effect of PFV infection on the expression of cellular genes. We found that the level of RelB mRNA, a member of the nuclear factor‐κB (NF‐κB) protein family, was significantly decreased as a result of PFV infection, and this was further confirmed with real‐time PCR. Interestingly, overexpression of RelB reduced PFV replication, whereas its depletion using small interfering RNA increased PFV replication. This inhibitory effect of RelB results from diminished transactivation of the viral long terminal repeat (LTR) promoter and an internal promoter (IP) by viral Tas protein. Together, these data demonstrate that PFV infection downregulates the viral inhibitory host factor RelB, which otherwise restricts viral gene expression.

## Graphical abstract

Foamy viruses (FVs) establish lifelong latent infections without evident pathology in the host. The roles of cellular factors in FV replication are poorly understood. In this study, we demonstrate that RelB interacts with PFV Tas, and RelB decreases Tas‐dependent transactivation of PFV gene expression in turn. PFV infection downregulates the viral inhibitory host factor RelB, which otherwise restricts viral gene expression.

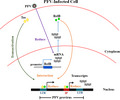

**Abbreviations**
APOBEC3: apolipoprotein B mRNA editing enzyme, catalytic polypeptide‐like 3
BFV: bovine foamy virus
BSA: bovine serum albumin
DAPI: 4′,6‐diamidino‐2‐phenylindole
FITC: fluorescein isothiocyanate
FVs: foamy viruses
HATs: histone acetyltransferases
HIV‐1: human immunodeficiency virus 1
HRP: horseradish peroxidase
HSV‐1: herpes simplex virus type 1
HTLV‐1: human T‐cell leukemia virus type 1
IFA: immunofluorescent assay
IFP35: IFN‐induced 35‐kDa protein
IP: internal promoter
LTR: long terminal repeat promoter
NF‐κB: nuclear factor‐κB
Nmi: N‐myc interactor
PBS: phosphate‐buffered saline
pcPFV: PFV full‐length infectious clone
PEI: polyethylenimine
PFV: prototype foamy virus
Pirh2: p53‐induced RING‐H2 protein
PML: promyelocytic leukemia protein
PVDF: polyvinylidene difluoride
RHD: Rel homology domain
RT: reverse transcription
SD: standard deviation
SDS/PAGE: polyacrylamide gel electrophoresis
shRNA: small hairpin RNA
TAD: transactivation domain
TREs: transactivation‐responsive elements
TRITC: tetramethyl rhodamine isocyanate
β‐Gal: β‐galactosidase

Foamy viruses (FVs), which belong to the *Spumaretrovirinae* subfamily of the *Retroviridae*, are complex retroviruses. These viruses have been isolated from different species, including primates, bovines, felines, and equines [1, 2, 3, 4, 5, 6, 7]. Although FVs have ubiquitous cellular tropism enabling them to infect a diverse range of cell types and to cause characteristic foam‐like cytopathic effects in culture system, they appear to be nonpathogenic in either naturally or accidentally infected hosts [8]. Unlike orthoretroviruses, FVs encode two functional promoters, the long terminal repeat (LTR) promoter and the internal promoter (IP). Tas, the main FV regulatory protein, is a DNA‐binding protein that can transactivate both promoters to enhance viral gene transcription by specifically binding to transactivation‐responsive elements (TREs) [9, 10].

To date, only a limited number of cellular factors have been found to modulate the replication of prototype foamy virus (PFV). For instance, the histone acetyltransferases (HATs) p300 and PCAF enhance Tas‐dependent transcriptional activation through specifically interacting with PFV Tas [11]; apolipoprotein B mRNA editing enzyme, catalytic polypeptide‐like 3 (APOBEC3) proteins can impair PFV reverse transcription (RT) by introducing fatal mutations into virus genome [12, 13]; tetherin inhibits virus release and PFV cell‐to‐cell transmission [14, 15]; promyelocytic leukemia protein (PML), N‐myc interactor (Nmi), and p53‐induced RING‐H2 protein (Pirh2) inhibit PFV replication through interacting with Tas [16, 17, 18]. To further identify cellular factors that are involved in the replication of PFV, we analyzed the transcriptomes of HT1080 cells infected by PFV and found significant reduction of RelB, a member of nuclear factor‐κB (NF‐κB) protein family.

In mammals, NF‐κB belongs to a class of dimeric transcription factors and plays a pivotal role in regulating the expression of various genes related to cell growth, apoptosis, inflammation, learning, memory, and immunity [19, 20]. The NF‐κB family consists of 5 members: RelA (p65), RelB, c‐Rel, NF‐κB1 (p50 and precursor p105), and NF‐κB2 (p52 and precursor p100). These proteins exist in the form of homodimers or heterodimers and can activate many target genes by binding to κB enhancers [21]. Although RelB belongs to the NF‐κB family, it is different from other two transcriptional active members RelA and c‐Rel in structure and function. In addition to the Rel homology domain (RHD) and the transactivation domain (TAD), RelB also has N‐terminal leucine zipper domain, which do not exist in other members of NF‐κB family [22]. RelB cannot form homodimer, but can form stable heterodimer with p50 and p52. RelB has no DNA‐binding activity and can only bind to target promoters through interacting with other proteins (e.g., p52, p50, and RelA). Compared with other members of the NF‐κB family, the biological action of RelB remains elusive.

Up to now, several viral proteins have been shown to promote viral replication or participate in viral pathogenesis by interacting with RelB. For example, bovine foamy virus (BFV) infection (BTas) upregulates the expression of RelB by activating the NF‐κB pathway in host cells and the increased amount of the RelB protein, in turn, enhances BFV transcription; human immunodeficiency virus 1 (HIV‐1) Tat activates NF‐kB pathway in two manners, and Vpr activates both canonical and noncanonical NF‐kB pathways that contribute to virus replication; human T‐cell leukemia virus type 1 (HTLV‐1) Tax1 stimulates RelB nuclear translocation by activating noncanonical NF‐κB pathway [23, 24, 25, 26].

In this study, we show that PFV Tas also associates with RelB; however, as opposed to the stimulating effect of RelB often seen in other viruses, the transactivation activity of PFV Tas is abrogated by RelB. To overcome this restriction function of RelB, PFV downregulates RelB to allow efficient viral gene expression.

## Materials and methods

### Plasmids and antibodies

DNA constructs pHA‐RelB and pQC‐RelB were described previously [26]. Maxine L. Linial (Division of Basic Sciences, Fred Hutchinson Cancer Research Center, Seattle, WA, USA) provided the pLTR‐luc [28], pIP‐luc [28], and pcPFV (PFV full‐length infectious clone) [29]. DNA clones PFV p3.1‐Tas and pFlag‐Tas were generated by inserting the region from nucleotide 9434 to nucleotide 10336 encompassing the *orf1* into the pcDNA3.1(+) (Invitrogen, Carlsbad, CA, USA) or pCMV‐Tag2B (Stratagene, La Jolla, CA, USA) vector.

Rabbit anti‐RelB antibodies were purchased from Cell Signaling Technology (Danvers, MA, USA). Mouse anti‐Flag (M2) and mouse anti‐HA antibodies were obtained from Sigma‐Aldrich (St. Louis, MO, USA). Mouse anti‐tubulin, rabbit anti‐HA, and horseradish peroxidase‐conjugated secondary antibodies were purchased from Santa Cruz Biotechnology (Santa Cruz, CA, USA). Fluorescein‐conjugated anti‐mouse and anti‐rabbit secondary antibodies were obtained from Jackson ImmunoResearch Laboratories (West Grove, PA, USA). DAPI (4′,6‐diamidino‐2‐phenylindole) and protein A beads were purchased from Sigma‐Aldrich. Antibodies against PFV Gag and PFV Tas were generated in BALB/c mice using bacterially purified PFV Gag (180‐433aa) and PFV Tas protein as immunogens. Because PFV Bet protein is a fusion protein produced by 88 amino acids at the 5' end of Tas and the complete Bel2 coding protein, this Tas antibody can be used to detect Bet protein. These polyclonal anti‐serums were used for western blotting and immunofluorescence imaging.

### Cell culture and transfection

HEK293T, HeLa, HT1080, and PFVL (BHK21‐derived indicator cells containing a luciferase gene under the control of the PFV LTR) [14, 27] cells were maintained in Dulbecco’s modified Eagle’s medium (Thermo Fisher, Waltham, MA, USA) supplemented with 10% fetal bovine serum (GE Healthcare, Cincinnati, OH, USA) in 5% CO_2_ at 37 °C. Plasmid transfections were performed by using polyethylenimine (PEI, Polysciences, Warrington, PA, USA) [28].

### Virus infections

The PFV stock was prepared by transfecting HEK293T cells with pcPFV DNA clone. To prepare cell‐free virus stocks, culture supernatants were cleared by low‐speed centrifugation (3000 g for 10 min), filtered through a 0.22‐μm‐pore‐size filter membrane, and kept at 4 °C. PFV titers were determined by infecting PFVL cells as described elsewhere [27].

Cells were infected with the PFV stock. The virus inoculum was washed off at 8 h postinfection. After additional 40 h, culture supernatants (500 μL) or infected cells (1 × 10^4^) were analyzed by incubating with PFVL cells (1 × 10^5^). The infected cells were also analyzed by western blotting using the indicated antibodies.

### Generation of stably transduced cell lines

To screen for cell lines that stably expressing RelB, we used a retroviral vector system. HEK293T cells were transfected with 1 μg pMLV‐Gag‐Pol, 0.5 μg pVSV‐G, and 1 μg pQC‐RelB DNA or vector. After 48 h, supernatants were collected and centrifuged at 1000 ***g*** to remove cell debris. HT1080 cells were infected with the harvested virus particles. After 48 h of transduction, cells were trypsinized and subcultured in selective medium containing 2 μg·mL^−1^ puromycin (Sigma‐Aldrich). The expression efficiency was assessed by western blotting using an antibody against RelB.

To screen RelB knockdown cell lines, small hairpin RNA (shRNA) targeting RelB was designed with the shRNA Sequence Designer (Clontech, Mountain View, CA, USA), and cloned into pSIREN‐RetroQ vector (Clontech). The target sequence for scramble shRNA (negative control, without a specific target in cells) was GAAGTAAGCGATATACATA, for RelB shRNA was GCAACATGTTCCCCAATCA (coding nucleotides 1664 to 1682). HEK293T cells were transfected with 1 μg pMLV‐Gag‐Pol, 0.5 μg pVSV‐G, and 1 μg pSIREN‐RetroQ DNA constructs. After 48 h, virus particles were harvested and transduced HeLa cells. Forty‐eight hours later, cells were trypsinized and subcultured in selection medium containing 2 μg·mL^−1^ puromycin. Knockdown efficiency was assessed by western blotting using an antibody against RelB.

### RNA‐seq and data analysis

HT1080 cells were infected by PFV (multiplicity of infection of 2) for 24 h or not. Total RNA from PFV‐infected and PFV‐uninfected HT1080 cells was extracted using TRIzol (Invitrogen, Carlsbad, CA, USA) reagent. The concentration and purity of RNA was tested by an ultraviolet spectrophotometer (NanoDrop). The value of OD260/OD280 ratio can be used as a reference of RNA purity. Our data of OD260/OD280 ratios were in the range from 1.9 to 2.1, which suggested that the total RNAs can be used for the succeeding experiment. Total RNA was submitted to the Beijing Genomics Institute (BGI) for sequencing. RNA‐seq was performed by using the Illumina HiSeq platform. The images generated by the sequencers were converted into nucleotide sequences by a base‐calling pipeline. The raw reads were saved in the fastq format, and we removed the dirty raw reads prior to analyzing the data. Three criteria were used to filter out dirty raw reads: remove reads with sequence adaptors; remove reads with more than 10% ‘N’ bases; and remove low‐quality reads, which have more than 50% QA ≤ 10 bases. All subsequent analyses were based on clean reads.

The reference sequences used were genome and transcriptome sequences downloaded from the UCSC website (version hg18). Clean reads were respectively aligned to the reference genome and transcriptome using HISAT [29]. The expression levels of the genes and transcripts were calculated by fragments per kilobase of transcript per million fragments mapped (FPKM) values. Using PossionDis [30], we identified differentially expressed genes between samples based on the following criteria: FDR ≤ 0.001 and fold change ≥ 2.

### Real‐time PCR

Cell RNA was extracted and reverse‐transcribed into cDNA. Real‐time PCR was detected by FastStart Universal SYBR Green PCR Master Mix (Roche, Basel, Switzerland) method. Relative RNA levels were normalized with GAPDH mRNA. The Real‐time primers are as follows: GAPDH forward (5’‐AACAGCGACACCCATCCTC‐3’) and GAPDH reverse (5’‐CATACCAGGAAATGAGCTTGACAA‐3’); RelB forward (5’‐CGCCCATCG CTTGTTCAT CGTG‐3’) and RelB reverse (5’‐CCGCAGCCCCAGCAGGTGTAT‐3’). The cycle parameters were 94 °C for 3 min, followed by 40 cycles at 94 °C for 30 s, 60 °C for 30 s, and 72 °C for 30 s. Fluorescence measurements were made for each cycle in a 72 °C step. The specificity of amplification was confirmed by melting curve analysis. 2^△△^ *^CT^* method was used for calculation.

### Luciferase reporter assay

HEK293T cells (2 × 10^5^) or HeLa cells (1 × 10^5^) were seeded in a 12‐well plate. After 24 h, reporter gene plasmid and pCMV‐β‐gal plasmid were transfected into the cells. Forty‐eight hours after transfection, luciferase assays were performed using the luciferase reporter assay system (Promega, Madison, WI, USA) according to the manufacturer’s instructions. β‐galactosidase (β‐gal) activity was used to normalize the transfection efficiency. Results of the luciferase reporter assays were the average of three independent experiments.

### Alu‐PCR

To detect the integration of PFV, HT1080 cells (2 × 10^5^) stably expressing RelB were seeded into 12‐well plates. As control, cells were treated with reverse transcriptase inhibitor AZT (10 μm) or integrase inhibitor raltegravir (10 μm) 2 h before PFV infection [31]. Cells were harvested 48 h after infection, and total DNA was extracted with DNeasy Blood and Tissue Kit according to the manufacturer’s instructions (Qiagen, Duesseldorf, Germany). The integrated PFV DNA was measured by semiquantitative PCR and real‐time PCR with Alu‐PCR primers. Alu‐PCR primers were as follows: ALU1 TCCCAGCTACTGGGGAGGCTGAGG, ALU2 GCCTCCCAAAGTGCTGGGATTACAG, SpA ATGCCACGTAAGCGAAACTT AGTATAATCATTTCCGCTTTCG, Lambda ATGCCACGTAAGCGAAACT, NestedR GAAACTAGGGAAAACTAGG. The semiquantitative PCR cycling parameters were 95 °C for 5 min; 95 °C for 30 s, 55 °C for 30 s, 68 °C for 3 min, 30 cycles, 68 °C for 7 min; and 4 °C heat preservation. Real‐time PCR was performed at 98 °C for 2 min and then 40 cycles at 98 °C for 10 s, 56 °C for 10 s, and 68 °C for 30 s. Finally, the melting curve was plotted.

### Co‐immunoprecipitation

For protein–protein interaction analysis, HEK293T cells (4 × 10^6^) were seeded into 10‐cm dish and then transfected with various plasmids. After 48 h, cells were harvested and lysed in immunoprecipitation buffer (50 mm Tris, pH 7.4; 150 mm NaCl; 2 mm EDTA; 3% glycerol; 1% NP‐40; complete, EDTA‐free protease inhibitor cocktail tablets). After sonication, cell lysates were centrifuged at 13 000 ***g*** for 10 min at 4 °C. Thereafter, supernatants were incubated with indicated antibodies (1 μg) for 3 h at 4 °C, and then rotated with Protein A‐agarose (Merck Millipore, Darmstadt, Germany) for 3 h at 4 °C. After being washed with lysis buffer for 6 times, the immunoprecipitation materials were boiled in 40 μL 2 × SDS loading buffer and subjected to western blotting using indicated antibodies.

### Western blotting

Cell lysates or immunoprecipitated materials were resolved by SDS/PAGE (polyacrylamide gel electrophoresis) and transferred onto the polyvinylidene difluoride (PVDF) membrane (GE Healthcare). The membranes were blocked with 5% nonfat milk, then incubated with primary antibodies at 4 °C overnight. After incubated with either goat anti‐rabbit or goat anti‐mouse secondary antibody conjugated with horseradish peroxidase (HRP) for 45 min, membranes were treated with enhanced chemiluminescence reagents (Merck Millipore, Darmstadt, Germany). Protein signals were detected by exposure to X‐ray films.

### Immunofluorescent assay

An indirect immunofluorescent Assay (IFA) was used to localize RelB (stained with rabbit anti‐HA antibody and fluorescein isothiocyanate (FITC)‐conjugated goat anti‐rabbit secondary antibody) and Tas (stained with mouse anti‐Flag antibody and tetramethyl rhodamine isocyanate (TRITC)‐conjugated goat anti‐mouse secondary antibody). HeLa cells (3 × 10^4^) were grown on a 12‐well plate and fixed with 4% paraformaldehyde in 1 × phosphate‐buffered saline (PBS) at room temperature for 10 min and permeabilized with 0.1% Triton X‐100 in 1 × PBS for 10 min at room temperature. After blocking with 3% bovine serum albumin (BSA) in 1 × PBS, cells were incubated with anti‐Flag or anti‐HA antibodies (1 : 100 dilution, 2 h at room temperature) and then incubated with fluorescein‐ and rhodamine‐conjugated secondary antibodies (1 : 100 dilution, 45 min at room temperature). Nuclei were stained with DAPI. Cells were fixed with 90% glycerol‐PBS and examined by Olympus X71 fluorescence microscope.

### Statistical analysis

Data were expressed as the mean ± standard deviation (SD) of the results of three independent experiments, in which each assay was in triplicate. Data were compared using the unpaired two‐tailed *t‐*test. *P* < 0.05 was considered significant (*P < 0.05, **P < 0.01, ***P < 0.001, ****P < 0.0001).

## Results

### Downregulation of RelB by PFV infection

**Fig. 1:**
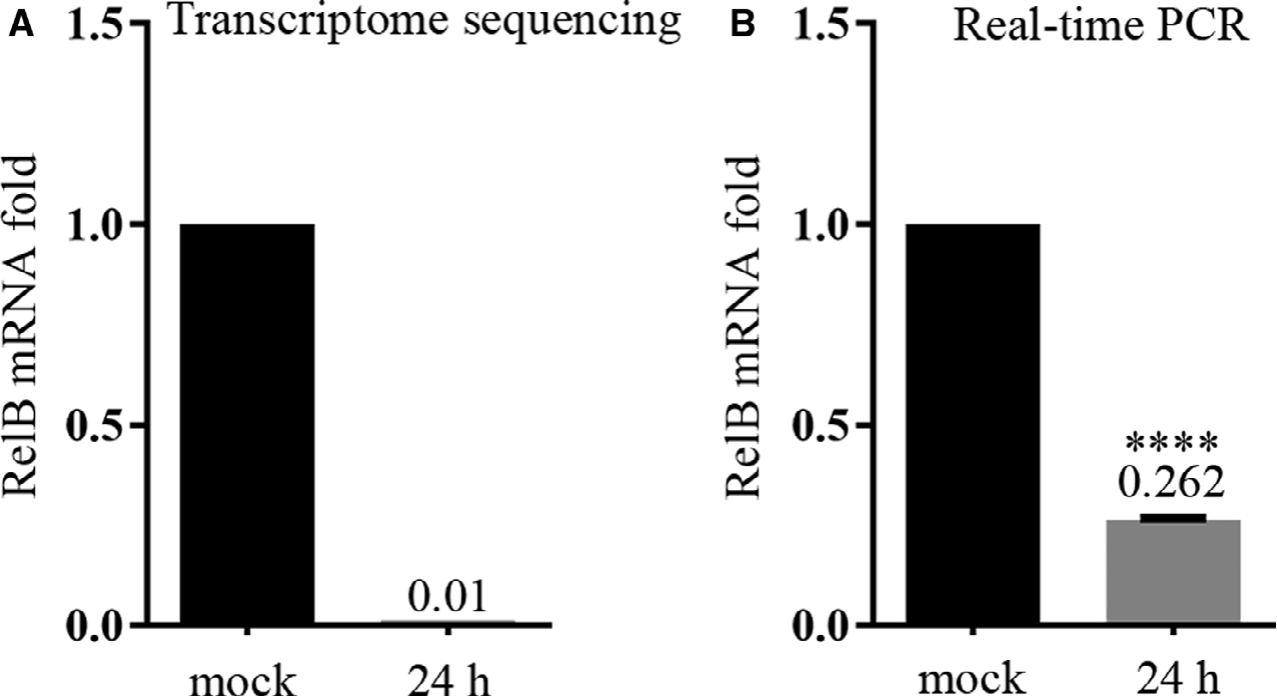
Downregulation of RelB by PFV infection. (A) After PFV infection of HT1080 cells for 24 h, RNA was extracted for transcriptome sequencing. (B) After HT1080 cells were infected with PFV for 24 h, the extracted RNA was reverse‐transcribed into cDNA, and the gene‐specific real‐time primers were designed for PCR to detect change of RelB mRNA levels. The results were the average of three independent experiments, and data were analyzed using GraphPad Prism software (paired *t‐*test, compared with mock, *P < 0.05, **P < 0.01, ***P < 0.001, ****P < 0.0001). Error bars indicate standard deviations.

To identify cellular factors involved in PFV replication, we determined the transcriptomes of the permissive cell HT1080 infected by PFV for 24 h in order to register changes in the levels of mRNA of cellular genes. PFV at a MOI of 2 can ensure that more than 50% of cells were infected based on our preliminary experiments (data not shown). Sequencing and data analysis were completed by the Beijing Genomics Institute. A total of more than 16 000 known mRNAs were detected. Compared with the mock‐infected group, PFV infection significantly upregulated the mRNA levels of about 290 genes and significantly downregulated the mRNA levels of about 100 genes. These data provide some clues for us to study the interaction between host and virus in the case of PFV infection. Analyzing the results, we noted that RelB, a member of NF‐κB protein family, was significantly downregulated by PFV infection (Fig. 1A), while other four members of the family, such as relA, c‐rel, p50, and p52, did not detect significant changes in transcriptional level 24 h after virus infection. Previous studies in our laboratory found that BFV infection (BTas) upregulates RelB expression in host cells and thus enhances BFV transcription. As a homologous virus, the effect of PFV infection on RelB is different from that of BFV, so we next explored the effect of RelB on PFV replication. First, we confirmed the decrease in RelB mRNA in PFV‐infected HT1080 cells by real‐time PCR (Fig. 1B).

### Overexpression of RelB inhibits PFV replication

**Fig. 2:**
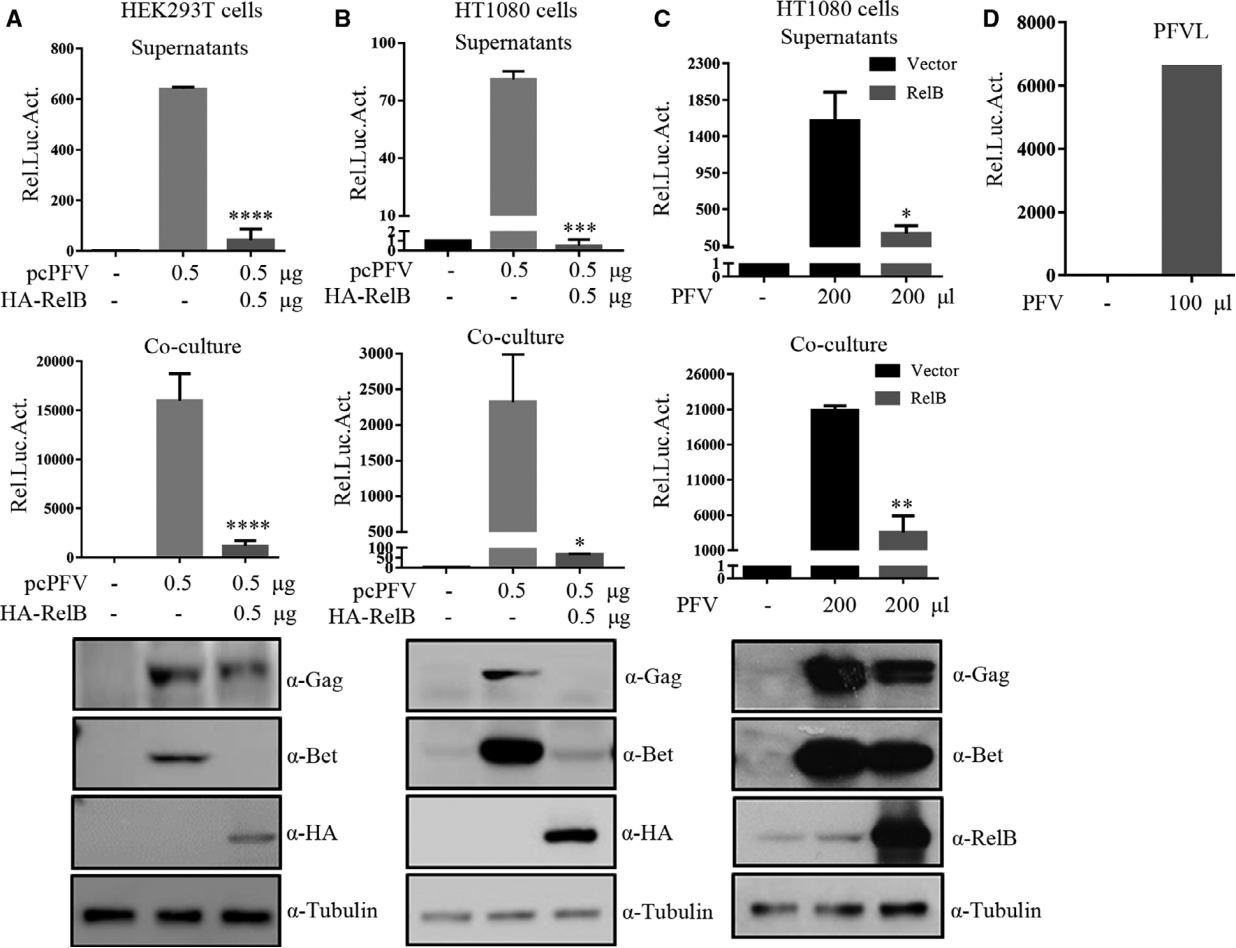
Overexpression of RelB inhibits PFV replication. A and B HEK293T cells (A) and HT1080 cells (B) were transfected with pcPFV (0.5 μg) and the empty vector or HA‐RelB (0.5 μg). Forty‐eight hours post‐transfection, supernatants (500 μL) or transfected cells (1/10 of total) were cocultured with PFVL indicator cell line for another 48 h and luciferase activity was measured then (compared with pcPFV, *P < 0.05, **P < 0.01, ***P < 0.001, ****P < 0.0001). The rest of cells were lysed and then immunoblotted with Gag, Bet, HA, and tubulin antibodies. (C) HT1080‐Control and HT1080 stably expressing RelB cells were infected with 200 μL of PFV whose titers had been determined using PFVL indicator cells (D). At 48 h postinfection, supernatants (500 μL) or infected HT1080 cell lines (1/10 of total) were cocultured with PFVL cells (compared with pQCXIP + PFV, *P < 0.05, **P < 0.01, ***P < 0.001, ****P < 0.0001). The infected HT1080 cell lines were also lysed and then immunoblotted with Gag, Bet, RelB, and tubulin antibodies. One‐way ANOVA was used to perform statistical test. All transfections were performed in triplicate. The results shown represent the averages of the results of three independent experiments. Error bars indicate standard deviations.

To investigate the effect of RelB on PFV replication, we transiently transfected full‐length infectious clone pcPFV and RelB in HEK293T cells. Forty‐eight hours post‐transfection, the culture supernatants (cell‐free PFV particles) or the transfected HEK293T cells (cell‐associated PFV particles) were incubated with the PFVL indicator cell line for another 48 h and luciferase activity was measured then. The rest of transfected HEK293T cells were collected for western blotting analysis. As shown in Fig. 2A, after overexpression of RelB, levels of both cell‐free and cell‐associated PFV were significantly reduced, and the expression levels of PFV Gag and Bet proteins in the transfected HEK293T cells were also significantly reduced compared with the control group. Similar results were obtained with transfection of HT1080 cells (Fig. 2B). These results demonstrated that RelB overexpression inhibited PFV replication.

In order to further confirm the inhibitory effect of RelB on PFV, we screened the HT1080 cell line stably expressing RelB by using a retroviral vector system, and infected the cell line with PFV virus stock solution. After 8 h, the medium was changed, and after 40 h of further infection, supernatants (cell‐free PFV particles) and cells (cell‐associated PFV particles) were incubated with the PFVL indicator cell line, and western blotting was used to detect the amount of viral proteins in the infected HT1080 cells (Fig. 2C). The titer of the virus used was shown in Fig. 2D. The results showed that the release of PFV and the level of intracellular replication in the cells stably expressing RelB were significantly lower than those in the control group, which confirmed the inhibitory effect of RelB on PFV replication.

### Knockdown of endogenous RelB promotes PFV replication

**Fig. 3:**
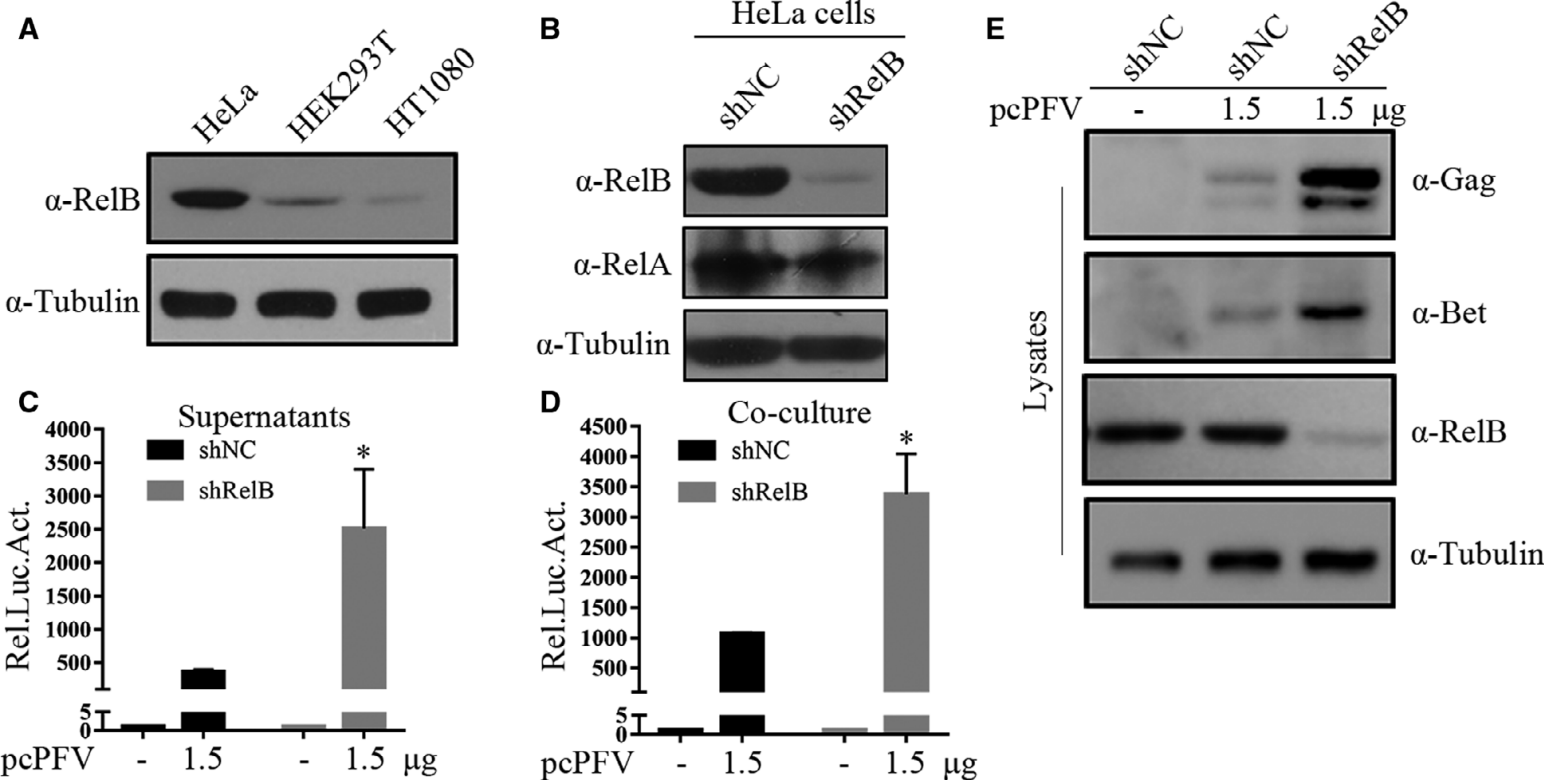
Knockdown of endogenous RelB promotes PFV replication. (A) Equal amount of cells (HEK293T, HeLa, and HT1080) were collected and lysed for western blotting analysis. (B) HeLa cells were infected with shRNA viral particles and negative control. Forty‐eight hours postinfection, cells were collected for western blotting analysis. (C–E) HeLa‐shControl and HeLa‐shRelB cells were transfected with pcPFV infectious clone (1.5 μg). Forty‐eight hours post‐transfection, C supernatants (500 μL) or D cells (1/10 of total) were cocultured with PFVL cells (compared with shControl + pcPFV, *P < 0.05, **P < 0.01, ***P < 0.001, ****P < 0.0001). (E) The rest of cells were collected for western blotting analysis. All transfections were performed in triplicate. One‐way ANOVA was used to perform statistical test. All transfections were performed in triplicate. The results shown represent the averages of the results of three independent experiments. Error bars indicate standard deviations.

Next, we explored the effect of endogenous RelB on PFV replication. We detected the expression of endogenous RelB protein in HEK293T, HT1080, and HeLa cells (Fig. 3A). Endogenous RelB in HeLa cells was much more than that in HEK293T cells and HT1080 cells. The HeLa cells were then generated with endogenous RelB knocked down. In order to determine the knockdown effect specifically targeting RelB, we also detected the expression of NF‐KB protein family member RelA in the knockdown cell lines (Fig. 3B). PFV replication increased in the RelB knockdown HeLa cells compared with that in the control cells (Fig. 3C,E), indicating that the endogenous RelB exerts an inhibitory effect on the replication of PFV.

### RelB downregulates the activation of PFV LTR and IP by Tas

**Fig. 4:**
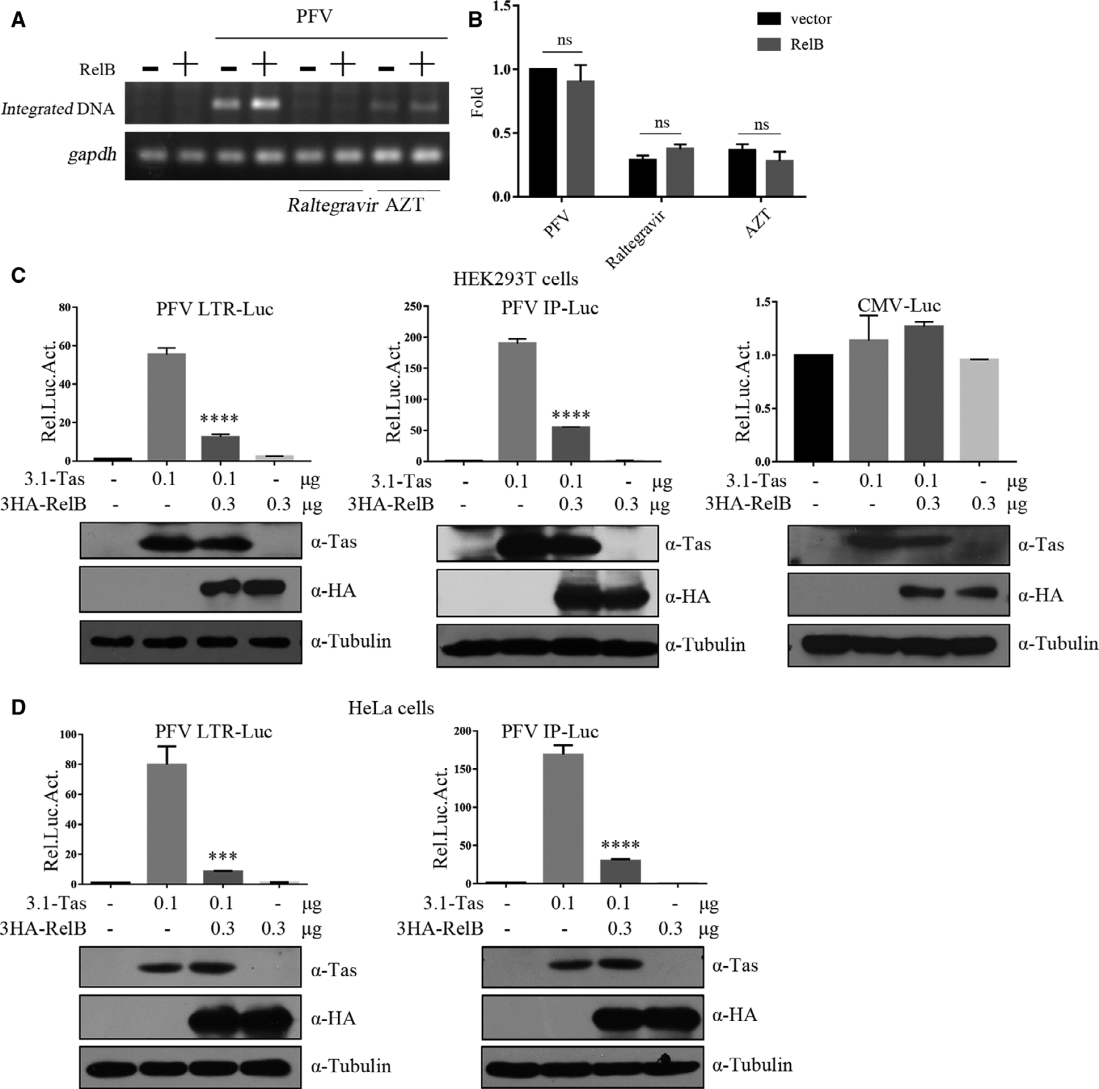
RelB downregulates the activation of PFV LTR and IP by Tas. (A and B) Levels of integrated proviral DNA were measured in semiquantitative PCR (A) and real‐time PCR (B) as described in Materials and Methods (two‐way ANOVA was used to perform statistical test). (C) HEK293T cells were transfected with LTR‐luc (0.025 μg), IP‐luc (0.01 μg), or CMV‐luc (0.025 μg) and combined with 3.1‐Tas (0.1 μg) and HA‐RelB (0.3 μg). To normalize transfection efficiency, pCMV‐β‐gal (0.025 μg) was cotransfected. Total DNA amounts were adjusted to 1 μg with an empty vector. At 48 h post‐transfection, luciferase activities were measured and corrected by β‐gal catalytic activities (one‐way ANOVA was used to perform statistical test. Compared with 3.1‐Tas, *P < 0.05, **P < 0.01, ***P < 0.001, ****P < 0.0001). The amount of Tas protein and RelB protein was detected by western blotting. (D) The same experimental process also repeated in HeLa cells. All transfections were performed in triplicate. The results shown represent the averages of the results of three independent experiments. Error bars indicate standard deviations.

To determine which step of PFV life cycle is inhibited by RelB, we first examined whether RelB inhibits the integration of PFV DNA. Based on the principle of Alu‐PCR, semiquantitative PCR and real‐time PCR were used to detect the integrated PFV genome. The results of semiquantitative PCR (Fig. 4A) and real‐time PCR (Fig. 4B) showed that overexpression of RelB did not affect PFV DNA integration in HT1080 cells, as opposed to the loss of PFV DNA integration with raltegravir (an integrase inhibitor) treatment.

We next tested whether RelB has any effect on PFV transcription. Tas is the only transcriptional activating factor of PFV, activating viral gene expression from both the 5’ LTR and the IP. We therefore utilized the luciferase reporter systems, controlled by the PFV LTR (LTR‐luc) or the IP (IP‐luc), to assess the effect of RelB on PFV gene transcription. The HEK293T cells were transfected with LTR‐luc or IP‐luc together with RelB in the presence of an empty or Tas‐expressing vector. The results showed that RelB reduced Tas transactivation of LTR and IP (Fig. 4C). With 300 ng of the RelB plasmid DNA, Tas transactivation of LTR and IP was inhibited by 77% and 71%, respectively. Meanwhile, RelB showed no effect on luciferase expression from the LTR‐luc or IP‐luc in HEK293T cells without Tas (Fig. 4C). Equal levels of Tas in the transfected cells were detected by western blotting. At the same time, we used the CMV promoter reporter plasmid to verify the specificity. The results showed that transfection of RelB or Tas alone and cotransfection of RelB and Tas had no effect on the basic transcription activity of the CMV promoter (Fig. 4C), which indicated that RelB had a specific effect on inhibiting Tas transactivation of PFV LTR and IP. Similar inhibitory effect by RelB on Tas was observed in HeLa cells (Fig. 4D).

### RelB interacts with Tas

**Fig. 5:**
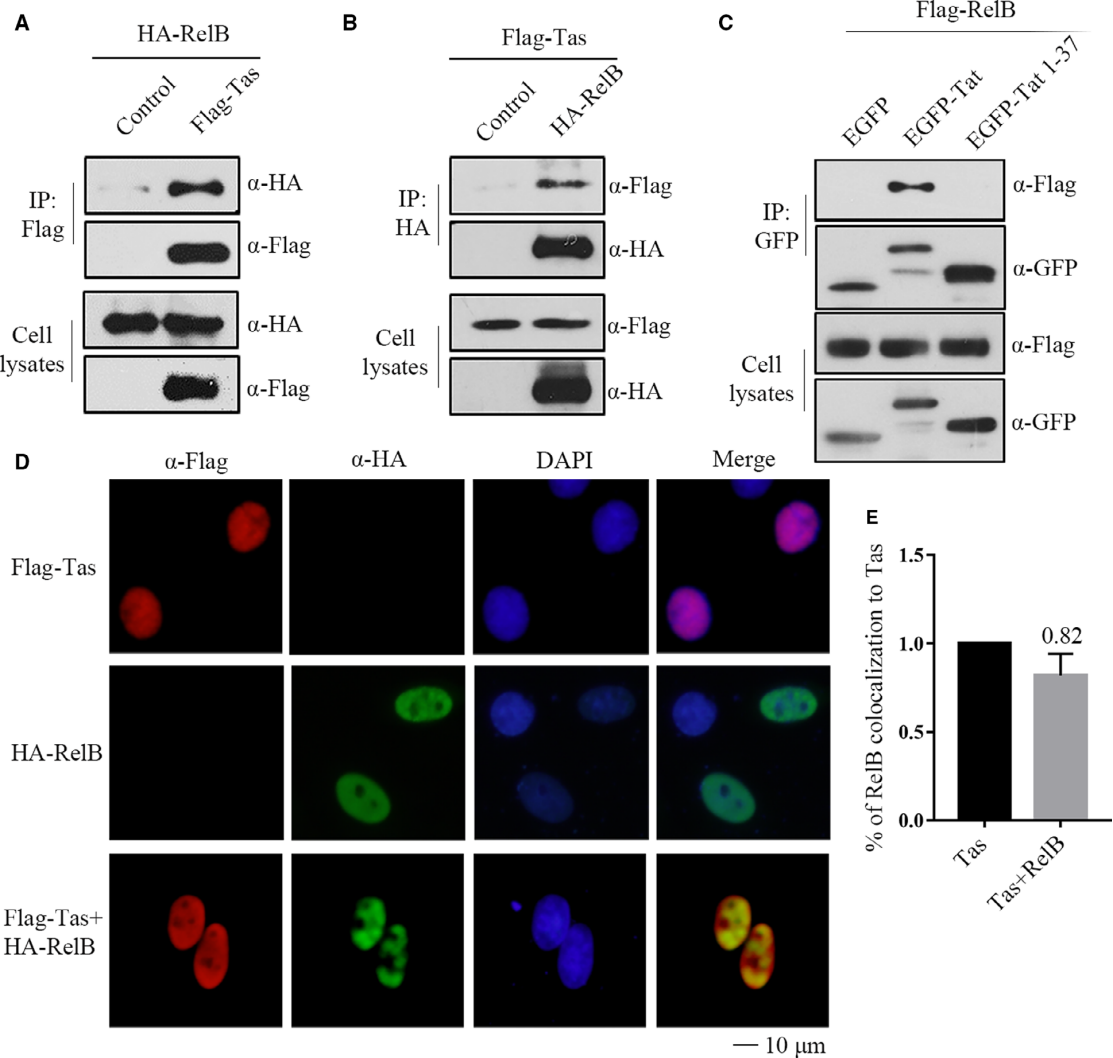
The RelB interacts with Tas. (A) HA‐RelB (4 μg) was transiently transfected into HEK293T cells together with the empty vector (control) or Flag‐Tas (4 μg, respectively). Co‐IP was performed with an anti‐Flag antibody. Samples from both cell lysates and immunoprecipitates were analyzed by western blotting using HA and Flag antibodies. (B) Flag‐Tas (4 μg) was transiently transfected into HEK293T cells together with the empty vector (control) or HA‐RelB (4 μg, respectively). Co‐IP was performed with an anti‐HA antibody. Samples from both cell lysates and immunoprecipitates were analyzed by western blotting using HA and Flag antibodies. (C) Flag‐RelB (4 μg) was transiently transfected into 293T cells together with the empty vector (control), Tat wild‐type (4 μg), or truncation mutant Tat 1‐37 (4 μg). Co‐IP was performed with an anti‐GFP antibody. Samples from both cell lysates and immunoprecipitates were analyzed by western blotting using Flag and GFP antibodies. (D) HeLa cells were transfected with Flag‐Tas (0.5 μg) or HA‐RelB (0.5 μg) or both. An indirect IFA was used to localize RelB (with rabbit anti‐HA antibody and FITC‐conjugated goat anti‐rabbit secondary antibody) and Tas (with mouse anti‐Flag antibody and TRITC‐conjugated goat anti‐mouse secondary antibody). Scale bar = 10 μm. (E) The percentage of RelB associated with Tas was quantified by counting the number of RelB‐positive cells, which are positive for Tas is shown. Between 30 and 35 images were analyzed. All transfections were performed in triplicate. The results shown represent the averages of the results of three independent experiments. Error bars indicate standard deviations.

We further tested whether RelB inhibits Tas through interaction in mammalian cells. Firstly, we performed co‐immunoprecipitation experiments to precipitate Flag‐Tas that was expressed together with HA‐RelB in HEK293T cells. The results of western blotting showed that HA‐RelB was precipitated together with Flag‐Tas (Fig. 5A). Similarly, we used the HA antibody to precipitate RelB and the Flag antibody to detect the presence of Tas protein in the enriched components (Fig. 5B). These results showed that RelB does interact with Tas in vivo. Our laboratory found that there is interaction between RelB and HIV‐1 Tat, and the core region of Tat is the key to the interaction. Tat truncation mutants without core region cannot coprecipitate to RelB [26]. Therefore, as a control, we also detected the interaction of RelB with wild‐type Tat and Tat 1‐37 truncated body lacking core region. In consistent with previous results, RelB interacted with full‐length Tat, but not with Tat 1‐37 truncated body (Fig. 5C). Indirect immunofluorescence staining showed that HA‐RelB and Flag‐Tas were colocalized within the nucleus (Fig. 5D). Colocalization of Tas and RelB was quantified (Fig. 5E), the results showed that Tas and RelB have strong colocalization. Together, these data show an association of Tas with RelB.

## Discussion

Viruses are obligate intracellular parasites and depend on cellular machinery for their efficient replication. For example, the NF‐κB pathway is hijacked by a number of viruses, such as HIV‐1, herpes simplex virus type 1 (HSV‐1), and BFV. Our previous studies showed that BFV infection upregulates cellular RelB expression through BTas‐induced NF‐κB activation, and RelB subsequently interacts with BTas and enhances transcription from the BFV LTR promoter [23]. In addition to BFV, HIV‐1 Tat can also use RelB as a cotransactivator to promote viral transcription [26]. However, different from BFV, PFV infection downregulates the expression of RelB to enhance the Tas‐induced LTR and IP transcription, facilitating the replication of PFV. Thus, compared with HIV‐1 and BFV, PFV has evolved another strategy to ensure effective viral transcription through reducing RelB.

Previous studies have reported that BFV Tas increases RelB expression through NF‐κB pathway [23]. Here, we show that PFV infection downregulates the expression of RelB, whereas other members of the NF‐κB pathway are not affected, but the molecular mechanism remains unclear. Unlike the effect on BTas, we found that RelB inhibited Tas‐mediated transactivation of PFV LTR and IP, and then inhibited PFV replication. Although RelB shows more flexibility in regulating gene expression, which can activate [22] and inhibit [32] the expression of target genes, we found that RelB did not affect the expression of Tas. Considering that no exclusive DNA‐binding activity of RelB had been discovered so far, and we found that there is an interaction between RelB and Tas, we speculate that the interaction between RelB and Tas may interfere with the binding ability of Tas to PFV promoters or the activation function of Tas.

Recently, some restriction factors have been found to inhibit FV replication by interfering with the function of Tas. For example, PML interacts directly with Tas and interferes with its ability to bind PFV LTR and IP [17]. IFP35 may inhibit BTas‐induced transactivation by interfering with the interaction of a cellular transcriptional activation factor(s) and BTas [41]. Nmi inhibits the Tas transactivation of PFV LTR and IP through interacting with Tas and sequestering it in the cytoplasm [18]. By interacting with Tas and downregulating its expression, Pirh2 negatively regulates Tas transactivation of PFV LTR and IP [16]. Similar to PML, IFP35, Nmi, and Prih2, RelB can also inhibit the Tas‐induced transactivation of PFV LTR and IP through interacting with Tas. However, the mechanism remains unclear.

In summary, our results demonstrate that RelB interacts with PFV Tas, and RelB decreases Tas‐dependent transactivation of PFV gene expression in turn. We propose a model to illustrate the interplay between PFV and RelB protein. Following PFV infection, RelB expression levels were decreased, which abolished the inhibitory effect of RelB on PFV Tas‐mediated transactivation on LTR and IP and facilitated the replication of PFV. Although our data show that RelB plays a key role in PFV transcription, it is not clear whether some other cellular components specifically participate in RelB‐inhibited Tas transactivation, which may function as inhibitors or stimulators of this process. Therefore, the identification of other components of the viral transcriptional complex will further enhance our current understanding of this virus–host cross talk.

## Conclusion

RelB belongs to the Rel protein family. It can interact with some viral proteins to promote viral replication and contribute to viral pathogenesis. In this study, we demonstrate that RelB interacts with PFV Tas, and RelB decreases Tas‐dependent transactivation of PFV gene expression in turn. PFV infection downregulates the viral inhibitory host factor RelB, which otherwise restricts viral gene expression. Our study found that RelB plays a different role in the process of prototype foamy virus infection than other viruses.

## Conflicts of interest

The authors declare no conflict of interest.

## Author contribution

JZ and JT conceived and designed the study, analyzed the data, and drafted the manuscript. JZ, CW, XT, and YX performed the experiments. KC contributed to data analysis. WQ supervised the project and participated in the design and interpretation of the study. All authors read and approved the final manuscript.

## Data Availability

The datasets used and/or analyzed during the current study are available from the corresponding author on reasonable request.
